# Water-soluble tomato concentrate modulates shear-induced platelet aggregation and blood flow *in vitro* and *in vivo*

**DOI:** 10.3389/fnut.2022.961301

**Published:** 2022-09-02

**Authors:** Lu Liu, Shunli Xiao, Yilin Wang, Yufang Wang, Lei Liu, Zhengxiao Sun, Qian Zhang, Xiaojie Yin, Fulong Liao, Yun You, Xuguang Zhang

**Affiliations:** ^1^Institute of Chinese Materia Medica, China Academy of Chinese Medical Sciences, Beijing, China; ^2^Byhealth Institute of Nutrition and Health, Guangzhou, China

**Keywords:** platelet function, shear induced platelet aggregation, vWF, inflammation, water-soluble tomato concentrate

## Abstract

Water-soluble tomato concentrate (WSTC), extracted from mature tomatoes, is the first health product in Europe that has been approved “to help maintain normal platelet activity to maintain healthy blood flow.” We hypothesized that WSTC might exert an influence on blood flow shear stress-induced platelet aggregation (SIPA) and in turn maintains healthy blood flow. We used a microfluidic system to measure the effects of WSTC on SIPA *in vitro.* We also used the strenuous exercise rat model and the κ-carrageenan-induced rat tail thrombosis model to demonstrate the effects of WSTC on blood flow. WSTC significantly inhibited platelet aggregation at pathological high shear rate of 4,000 s^–1^ and 8,000 s^–1^ *in vitro* (*P* < 0.05 or *P* < 0.01). WSTC reduced the platelet adhesion rate and increased the rolling speed of platelets by inhibiting binding to Von Willebrand Factor (vWF) (*P* < 0.05 or *P* < 0.01). The oral administration of WSTC for 4 weeks in strenuous exercise rats alleviated hyper-reactivity of the platelets and led to a significant reduction in the plasma levels of catecholamine and IL-6. WSTC treatment also led to a reduction in black tail length, reduced blood flow pulse index (PI) and vascular resistance index (RI), and ameliorated local microcirculation perfusion in a rat model of thrombosis. WSTC exerted obvious inhibitory effects on the platelet aggregation induced by shear flow and alleviated the blood flow and microcirculation abnormities induced by an inflammatory reaction.

## Introduction

Arterial thrombosis, as a significant cause of myocardial infarction (MI) and ischemic stroke, results in more than 14 million deaths annually, and also represents the leading cause of death and disability globally (1). Virchow’s triad of thrombosis formation was postulated in the nineteenth century, including vascular injury, blood flow abnormalities, and blood hypercoagulability. Over recent years, a significant body of research has focused on abnormalities in the endothelium, platelets, and blood flow during the formation of thrombi. Endothelial monolayer dysfunction may lead to vascular injury in response to chemical and mechanical stimuli, while disturbed blood flow patterns (which can occur naturally or are associated with vascular injuries) can cause thrombosis. Platelets in individuals with obesity, hypertension, and insulin resistance showed increased activity at baseline in response to mechanical and chemical agonists, ultimately leading to robust aggregation (1).

Platelets are not only activated by specific chemicals (such as ADP, collagen, arachidonic acid, etc.) but also have a strong sensitivity to blood flow shear stress (2). Blood flow in the vascular system is typically characterized by two key variables: shear stress and shear rate. Shear stress is the force per unit area applied between adjacent layers of fluid, and shear rate (units of inverse time) is the relative velocity gradient between adjacent layers of fluid. The maximum value of the shear rate is located at the vessel wall. And the wall shear rates generally range from about 10 s^–1^ in large veins to 1,000–2,000 s^–1^ in arteries, the corresponding wall shear stresses are typically 0.35–70 dynes/cm^2^ in the normal vasculature. Wall shear rates > 4,000 s^–1^ are generally considered pathologic, whereas maximal wall shear rates up to 40,000 s^–1^ have been described for severe atherosclerotic arteries (3, 4). If blood flow in a normal vessel increases, for example, due to exercise, the wall shear rate increases proportionally with the flow rate. A significant increase in shear stress was observed at high-intensity exercise in the carotid artery, from resting with 26.5 ± 3.3 dynes/cm^2^ to high-intensity cycling with 77.5 ± 20.3 dynes/cm^2^ (5). A high-flow shear stress can directly activate platelets and lead to aggregation (6). Shear-induced platelet aggregation (SIPA) is an important factor in arterial thrombosis and represents a contributing factor to the atherothrombotic occlusion of blood vessels. High shear stress is responsible for extensive conformational transitions in Von Willebrand Factor (vWF) that change their structure from a globular form to a stretched linear conformation that induces platelet aggregation in a manner that is dependent on vWF-glycoprotein (GP) Ib interactions (7). Antiplatelet drugs, such as aspirin and clopidogrel, exert limited effects on thrombosis caused by high shear stress (8, 9), and the risk of bleeding also increases during the clinical administration of these drugs.

Although moderate exercise is associated with an overall decreased risk of cardiovascular diseases, it has been reported that moderate exercise elevates blood flow-inducing shear stress which is vascular protective and therefore may overcome sedentary-related decline in vascular function. However, high-intensity exercise or strenuous exercise may induce abnormal high blood flow shear stress and flow disturbances which contributes as a factor to platelet aggregation; therefore, high-intensity exercise may increase the risk of vascular thrombotic and cardiac events (10, 11). The second consequence of platelet activation during exercise is the promotion of inflammation, and this event manifests as increased levels of catecholamine and IL-6 (12).

κ-carrageenan, a sulfated polysaccharide extracted from seaweed, is used to prepare multiple tissue inflammation models such as the rat tail thrombosis model (13–15). The rat tail thrombosis model induced by κ-carrageenan is closely related to vascular endothelial inflammation and blood vessel injury. It is presumed that carrageenan induces endothelial inflammation, thus leading to the production of inflammatory factors, such as IL-6 and TNF-α. The excessive production of inflammatory factors and blood flow (16) disturbs the homeostatic balance and may cause thrombosis.

Tomato (*Lycopersicon esculentum*), one of the main vegetables in the Mediterranean diet, contains a variety of ingredients that are beneficial to human health, including flavonoids, phytosterols, carotenoids, and several water-soluble vitamins and minerals (17). A recent study showed that the consumption of tomato juice has the potential to increase the levels of antioxidant enzymes in erythrocytes and reduce the serum levels of malondialdehyde in overweight and obese females (18). Water-soluble tomato concentrate (WSTC), extracted from mature tomatoes, is a natural food product that can exert cardioprotective effects by influencing antiplatelet function and contains nucleosides, phenolic derivatives, and flavonoid derivatives (19, 20). WSTC is the first health product in Europe that has been approved under Article 13 (5) of the European Health Claims Regulation “to help maintain normal platelet activity to maintain healthy blood flow.” Research demonstrated that WSTC efficiently prevented platelet aggregation in response to ADP, collagen, and thrombin (21). However, there is a clear lack of direct and clear evidence relating to the effects of WSTC on shear stress-induced platelet aggregation (SIPA).

This study focused on the antiplatelet activity of WSTC in terms of mechanic force stimuli, we hypothesized that WSTC might exert influence on shear SIPA and anti-inflammatory, and thus helped to maintain a healthy blood flow. A high-intensity exercise rat model was used to demonstrate the possible action of WSTC on SIPA *in vivo*; furthermore, we used the rat tail thrombosis model to demonstrate the effect of WSTC on blood flow. We found that WSTC reduced high shear stress-induced platelet activation by blocking the interaction between vWF and platelet integrin GP Ib. Our results implied that WSTC can inhibit platelet hyperactivity and maintain normal blood flow by modifying platelet responses to mechanic force stimuli.

## Materials and methods

### Reagents

The following reagents were used in the present study: WSTC (BY-Health Co., Ltd., Batch No. 201701), κ - Carrageenan (Type I, Ekasion, Israel, C1013), trisodium citrate dihydrate (Sinopharm Chemical Reagent Co., Shanghai, China, 20140306), calcein-AM (Invitrogen, Carlsbad, United States, C3100MP), Collagen (Chronolog, Pennsylvania, United States, 385), vWF (Hematologic Technologies, Vermont, United States, HCVWF-0190), ADP (Beijing Techlink Biomedical Technology Co., Ltd., Beijing, China, 20180806A), and aspirin (Bayer Health Care AG, Leverkusen, Germany, 1806210) We also used a rat catecholamine ELISA Kit (BioVision, California, United States, 7F03E44630), a rat vWF ELISA Kit (Shanghai Enzyme-Linked Biotechnology Co., Shanghai, China, 202109), a rat IL-6 ELISA kit (Dakewe biotechnology, Shenzhen, China, 2207-1), Prothrombin time (PT) (lot, 200803901), activated partial thromboplastin time (APTT) (lot 200809200), thrombin time (TT) (lot, 200709501), and fibrinogen (FIB) (lot 210304200), which were purchased from Shanghai Changdao Biotechnology Co., Ltd., Shanghai, China.

### Platelet preparation

Rats were obtained from the Experimental Animal Centre, Charles River Laboratories (Beijing, China) (SCXK2016-0006) and housed at 15^°^C–28^°^C with a humidity of 45–55%. The rats were given food and water *ad libitum* for 3 days before use. Blood was withdrawn from male Sprague-Dawley rats (weighing 250–270 g) using protocols that were carried out in accordance with the guidelines approved by the Animal Care and Use Committee of Institute of Chinese Materia Medica, China Academy of Chinese Medical Sciences, Beijing, China (Ethics approval number: 2021B133). In brief, rats were anesthetized by injection with 1% sodium pentobarbital (60 mg⋅kg^–1^); then an 8-mL sample of whole blood was withdrawn from the abdominal aorta. Each sample was anti-coagulated with 109 mM sodium citrate (1:9). For platelet aggregation assays, platelet-rich plasma (PRP) was obtained by centrifugation of the blood sample at 200 × g for 10 min.

### Shear-induced platelet aggregation assay *in vitro*

High Shear BioFlux 48-well plates (Fluxion Biosciences, Alameda, CA, United States) were used to measure SIPA as described previously (22). In brief, the microfluidic channels were coated with 40 μg⋅mL^–1^ collagen for 1 h and then blocked with 0.5% bovine serum albumin (BSA) in phosphate-buffered saline (PBS) at room temperature. The platelet density in PRP was adjusted to 5.0 × 10^10^⋅L^–1^ with Tyrode’s solution and the platelet suspension was then incubated with calcein-AM (5 μM) for 1 h at room temperature in the dark. Calcein-AM-labeled platelets were treated with WSTC (0.6 g⋅L^–1^, 2 and 6 g⋅L^–1^) or vehicle (saline) for 10 min at 37^°^C in the dark.

Platelets were perfused in the microfluidic channels with incremental pressures of 0.13, 0.33, 0.66, 1.32, and 2.64 psi for 5 min; this resulted in wall shear rates of 400, 1,000, 2,000, 4,000, and 8,000 s^–1^ for 25 min. Fluorescent micrographs of the adhesion and aggregation of platelets were captured by a time-lapse inverted microscope (wavelength FITC module; exposure time: 400 ms) using an Axio Observer 7 (Carl Zeiss AG, Oberkochen, Germany) and a C11440-42U30 digital camera (Hamamatsu Photonics, Shizuoka, Japan). The BioFlux-1000z system (Fluxion Biosciences, Alameda, CA, United States) (Supplementary Figure 1) was utilized to control the shear rate and image acquisition settings. Platelet aggregation rates were determined by fluorescence intensity using the BioFlux Montage software (Fluxion Biosciences, Alameda, CA, United States). Three independent experiments were carried out.

### Platelet adhesion and rolling assays under shear stress

Platelet adhesion and rolling assays were carried out with High Shear BioFlux 48-well plates. The microfluidic channels were coated with 70 μg⋅mL^–1^ vWF for 1 h and then blocked with 0.5% (v/v) BSA in PBS for 10 min at room temperature. Sodium citrate anti-coagulated whole blood was treated with WSTC (0.6, 2.0, and 6.0 g⋅L^–1^) or vehicle (saline) for 10 min at 37°C before perfusions at a shear rate of 750 s^–1^ (shear stress of 3 Pa). Subsequently, the channels were washed for 5 min at a shear rate of 1,000 s^–1^ with Hank’s Balanced Salt Solution (HBSS) (with calcium and magnesium) until all red and white blood cells were clear of channels. Only platelets were loaded on the surface of channels coated with vWF protein. The rolling of platelets under a shear rate of 6,000 s^–1^ with HBSS containing saline or WSTC (0.6, 2.0, or 6.0 g⋅L^–1^) was recorded by time-lapse imaging every second for 30 s. Platelet adhesion and rolling speed were measured using the BioFlux Montage software (Fluxion Biosciences, Alameda, CA, United States). Measurements of distance/time were taken for 10 rolling platelets in each field. Three independent experiments were carried out, as described above. The adhesion inhibitory rate was calculated as follows: (platelet adhesion number at the beginning of flow—platelet adhesion number after 10 s flow)/platelet adhesion number at the beginning of flow × 100%.

### Animals

All animal care and experimental procedures were approved by the Animal Care and Use Committee of Institute of Chinese Materia Medica, China Academy of Chinese Medical Sciences (Beijing, China), with ethics approval No. 2021B133. In total, 120 male Sprague Dawley (SD) rats were obtained from the Experimental Animal Centre, Charles River Laboratories (Beijing, China) (SCXK2016-0006), weighing 150–170 g. All rats were bred and maintained under specific pathogen-free conditions at the Institute of Basic Theory (China Academy of Chinese Medical Sciences, Beijing, China) with a controlled temperature (22 ± 2^°^C) and humidity (60 ± 10%) and a 12-h light/dark cycle. Before the experiments, the animals were acclimated for 5 days under controlled laboratory conditions. All animals were given drinking water *ad libitum* and were maintained on a normal pellet diet, the diet component of rats contained corn, soybean meal, fish meal, flour, bran, salt, calcium hydrogen phosphate, multivitamins, multiple trace elements, and amino acid, respectively (Keao Heli Feed Co., Beijing, China, 21083213). The animals were randomly divided into different treatment groups. Rats were weighed weekly (Supplementary Table 1). Rats were given oral WSTC at doses of 25, 75, and 150 mg⋅kg^–1^ or Aspirin at a dose of 0.75 mg⋅kg^–1^. Both the normal and model groups of rats received the same volume of distilled water.

### The rat model of strenuous exercise

The rats were given three doses of oral WSTC (25, 75, and 150 mg⋅kg^–1^) daily for 30 days; the swimming training started on Day 20th. To familiarize the rats with water immersion and reduce water-induced stress, the rats were trained to swim for 20 min daily for 3 days from D20th to D22nd. After the rats had adapted to the swimming exercise, they were subjected to strenuous swimming with a weight equivalent to 5% of their body weight tied to their tails for 7 days (D23th to D29th). The swimming training was conducted in a container (length: 100 cm; width: 60 cm; depth: 80 cm) with the water temperature maintained at 22 ± 2^°^C. Exhaustion was determined by two criteria: the rats remained below the water surface for 10 s or the rats showed a lack of movement when placed on a flat surface.

On Day 30th, 1 h after WSTC administration, the rats in each group were anesthetized by an intraperitoneal injection of 60 mg⋅kg^–1^ of 1% sodium pentobarbital; blood samples were then taken from the abdominal aorta; and anti-coagulation involved 109 mM sodium citrate (1:9).

#### Platelet aggregation assay *ex vivo*

For platelet aggregation assay, PRP was obtained following centrifugation of blood samples at 200 × g for 10 min, and platelet-poor plasma (PPP) was obtained by centrifugation of the residue component at 2,000 × g for 10 min. PPP was used to adjust the platelet concentration in PRP to 4 × 10^8^ platelets per milliliter. The platelet maximal aggregation rate was then assessed by ADP (2.5 μM) stimulation using an aggregometer (LBY-NJ4, Techlink Biomedical Group, Beijing, China). The rate of inhibition was calculated as follows: (maximal aggregation rate in control group—maximal aggregation rate in WSTC group)/maximum aggregation rate in the control group × 100%.

#### Determination of the levels of Von Willebrand Factor, catecholamine, IL-6, and coagulation function

Blood samples were centrifuged at 4,000 × g for 20 min and 250 μL of upper plasma was used to determine the levels of vWF, catecholamine, and IL-6 by enzyme-linked immunosorbent assay (ELISA) kits; these kits were used in accordance with the manufacturer’s instructions. The levels of vWF, IL-6 and catecholamine were calculated by the optical density (OD at 450 nm wavelength) value of each sample. Plasma coagulation function was evaluated by measuring TT, PT, APTT, and FIB with a Coagulation Analyzer (C3100, PRECIL, Beijing, China).

### Carrageenan-induced rat tail thrombosis model

κ-carrageenan has previously been used to induce rat tail thrombosis to investigate the effect of WSTC on platelet aggregation and on the maintenance of normal blood flow *in vivo*. Our preliminary study optimized the κ-carrageenan dose, environment temperature, and infarction time. The optimized dose of κ-carrageenan was 1 mg⋅kg^–1^ BW and the environment temperature was 18 ± 1^°^C. In addition, the total length of the tail of each rat needed to exceed 13 cm.

Three doses of WSTC (25, 75, and 150 mg⋅kg^–1^) or aspirin (7.5 mg⋅kg^–1^) were orally given to the rats daily as an intervention or positive control, respectively, for 28 days, and the induction of thrombosis was conducted on Day 26th. The general procedures were described previously (16, 23). In brief, κ-carrageenan was dissolved in saline and injected into the dorsal tail vein at a dose of 1 mg⋅kg^–1^. The frequency of infarction and the length of the infarcted region in the tail were recorded at 2, 6, 24, and 48 h after κ-carrageenan injection. The control group was administered with only saline. Redness, swelling, and a dark auburn color were observed in the tail within 2–3 h after the intravenous injection of κ-carrageenan, thus indicating that thrombosis had formed in the tail. The length of infraction in the tail increased with the time elapsed and became stable 6 h after κ-carrageenan injection at a room temperature of 18^°^C. The black tails were photographed after 24 h.

#### Blood flow and microcirculation perfusion assays

On Day 27th, we detected the blood flow in the femoral artery of each rat with a high-frequency ultrasound system (Vevo 2100, VisualSonics, Toronto, Ontario, Canada), as described previously (24). In brief, rats were anesthetized with an intraperitoneal injection of 50 mg⋅kg^–1^ of 1% sodium pentobarbital. We also used hair removal cream to remove fur from the hind limbs to expose the skin. A probe with a 30-MHz transducer was placed close to and parallel to the femoral artery under B-mode and the x-axis and y-axis were adjusted under PW-mode. Next, we determined the femoral artery diameter (D), peak systolic blood flow velocity (PSV), end-diastolic blood flow velocity (EDV), mean blood flow velocity (MV), pulse index (PI), and vascular resistance index (RI).

A ZR-Pericam PSI laser speckle flow imaging system (PERIMED, Sweden) was used to detect microcirculation perfusion, as described previously (25). Rats were anesthetized and body temperature was maintained at 36^°^C–37^°^C by an animal electric blanket (Harvard Medical Co., United States). Visualization of the microcirculation perfusion in the hind limb, plantar, and tail was performed by a recording system (Pimsoft, PERIMED, Sweden). Laser Doppler flowmeter signals were recorded in arbitrary blood perfusion units.

#### Platelet aggregation assay *ex vivo* in a tail thrombosis model

On Day 28th, 1 h after the administration of WSTC, the rats in each group were anesthetized by an intraperitoneal injection of 1% sodium pentobarbital (60 mg⋅kg^–1^). Blood was then taken from the abdominal aorta and anti-coagulated using 109 mM sodium citrate (1:9). Then, platelet aggregation was detected as described in section “The rat model of strenuous exercise.”

#### Histological examination

Finally, the rats were euthanized by an overdose of anesthetic following the intraperitoneal injection of sodium pentobarbital. Next, 1 cm tail tissue was cut from an area of 6 cm from the tip of the tail to prepare pathological sections. Histological changes in the tail tissue following WSTC treatment were observed by microscopy and the area of thrombosis was analyzed. Pathological changes associated with tail thrombosis were graded and scored.

### Statistical analysis

All data are shown as mean ± standard error of the mean (S.E.M). The effect of WSTC on SIPA *in vitro* was analyzed by two-way analysis of variance (ANOVA). Other data were subjected to one-way ANOVA followed by Duncan’s multiple range tests to analyze the difference between groups. All statistical analysis was performed with Prism 9.3.1 software (GraphPad Software, LLC). In all cases, *P* < 0.05 was considered to indicate statistical significance.

## Results

### Effects of treatment with water-soluble tomato concentrate on shear-induced platelet aggregation *in vitro*

**FIGURE 1:**
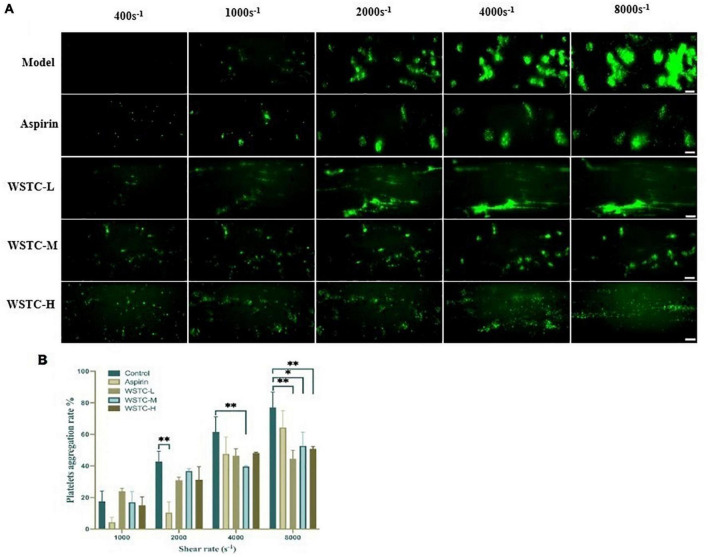
Effects of water-soluble tomato concentrate (WSTC) on shear-induced platelet aggregation. **(A)** Representative images showing effects of Model group and groups treated with WSTC at 0.7 g⋅L^–1^ (WSTC-L), 2.0 g⋅L^–1^ (WSTC-M), or 6.0 g⋅L^–1^ (WSTC-H) and Aspirin (1 mM) on platelet aggregation under shear rates of 400, 1,000, 2,000, 4,000, and 8,000 s^–1^. The scale bar is 50 μm. **(B)** Mean platelet aggregation rate at the end of exposure indicated shear rates under the conditions shown in **(A)**. *P < 0.05 and ^**^P < 0.01, compared to control group, analyzed by two-way ANOVA test.

First, we investigated whether WSTC affected SIPA. SIPA was induced by applying shear to platelets on the surface of collagen-coated microfluidic channels, starting at a shear rate of 400 s^–1^ and increasing to 1,000 and 2,000 s^–1^ observed under physiological flow conditions. Shear rate was increased further to 4,000 and 8,000 s^–1^ under high pathological flow conditions. We identified an interaction between shear rate and WSTC treatment on platelet aggregation by two-way ANOVA (*F* = 5.801, *P* < 0.001), thus, indicating that WSTC had a significant effect on SIPA. As shear stress increased, the platelet aggregation rate in the model group increased significantly. Treatment with WSTC at a concentration of 0.7 g⋅L^–1^ (WSTC-L), 2.0 g⋅L^–1^ (WSTC-M), and 6.0 g⋅L^–1^ (WSTC-H) strongly reduced the platelet aggregation induced by a high shear rate of 4,000 s^–1^ and 8,000 s^–1^ (Figure 1A).

Figure 1B shows the mean platelet aggregation rate at shear rates of 1,000, 2,000, 4,000, and 8,000 s^–1^ under the indicated conditions. Compared with the vehicle group, treatment with WSTC inhibited the platelet aggregation induced by a high pathological shear rate of 4,000 s^–1^ and 8,000 s^–1^. The inhibitory rates of platelet aggregation were 42.1 ± 7.1%, 31.6 ± 11.2%, and 34.0 ± 2.0% under WSTC treatment at concentrations of 0.7, 2.0, and 6.0 g⋅L^–1^, respectively, under the shear rate of 8,000 s^–1^ (*P* < 0.01 or *P* < 0.05). Meanwhile, 1 mM aspirin treatment only inhibited platelet aggregation under the physiological shear rate of 2,000 s^–1^ (Figure 1B).

### Effects of water-soluble tomato concentrate on Von Willebrand Factor-induced platelet adhesion and rolling under shear stress

**FIGURE 2:**
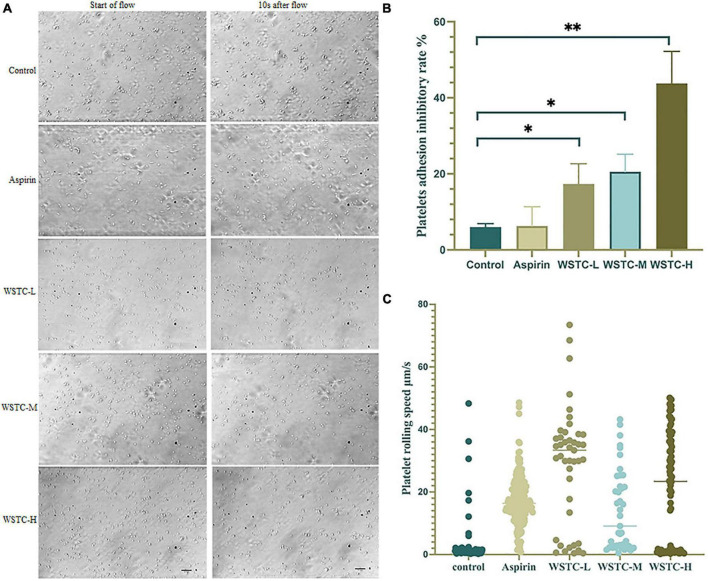
Effects of water-soluble tomato concentrate (WSTC) on Von Willebrand Factor (vWF)-mediated platelet adhesion and rolling velocity under shear stress. **(A)** Representative images showing effects of vWF-mediated platelet adhesion under 6 Pa shear stress in each group, the first image of each row was taken at the beginning of the flow, and the second image shown is at 10 s after flow. The scale bar is 20 μm. **(B)** Mean platelet adhesion inhibitory rate of the control group, WSTC-L, WSTC-M, WSTC-H, and Aspirin group under the conditions shown in **(A)**, the inhibitory rate was calculated as follows: (adhesion platelet numbers at beginning − adhesion platelet numbers after 10 s shear flow)/adhesion platelet numbers at beginning × 100%. *P < 0.05, compared to control group; ^**^P < 0.01, compared to control group. **(C)** Mean platelet rolling speed in the control group, WSTC-L, WSTC-M, WSTC-H groups, and Aspirin group under conditions shown in **(A)**.

The adhesion and rolling of platelets to vWF proteins is the initiation stage of SIPA. Figure 2A shows that under a high flow shear rate of 6,000 s^–1^, WSTC inhibited platelet adhesion by blocking the binding of integrin GP Ibα to vWF protein. Compared with the model group, WSTC at concentrations 0.7, 2, and 6 g⋅L^–1^ significantly inhibited the platelet adhesion induced by vWF under the shear rate of 6,000 s^–1^ duration of 10 s (*P* < 0.01, *P* < 0.05) (Figure 2B). The reduced adhesion of platelets on the vWF protein layer due to the WSTC treatment resulted in a significantly higher mean rolling speed (WSTC-L: 29.05 ± 18.55 μm⋅s^–1^, WSTC-M: 14.12 ± 12.90 μm⋅s^–1^, and WSTC-H: 21.04 ± 17.81 μm⋅s^–1^, respectively) when compared to the control group (3.2 ± 7.73 μm⋅s^–1^) (Figure 2C). Collectively, these results suggested that WSTC might inhibit the binding of platelet’s integrin GP Ibα to vWF protein, thereby increasing the rolling speed of platelets and thus reducing the adhesion rate and the risk of platelet aggregation induced by high shear.

### Effects of water-soluble tomato concentrate on platelet aggregation and the levels of plasma catecholamine, IL-6, and Von Willebrand Factor in strenuous exercise rats

**FIGURE 3:**
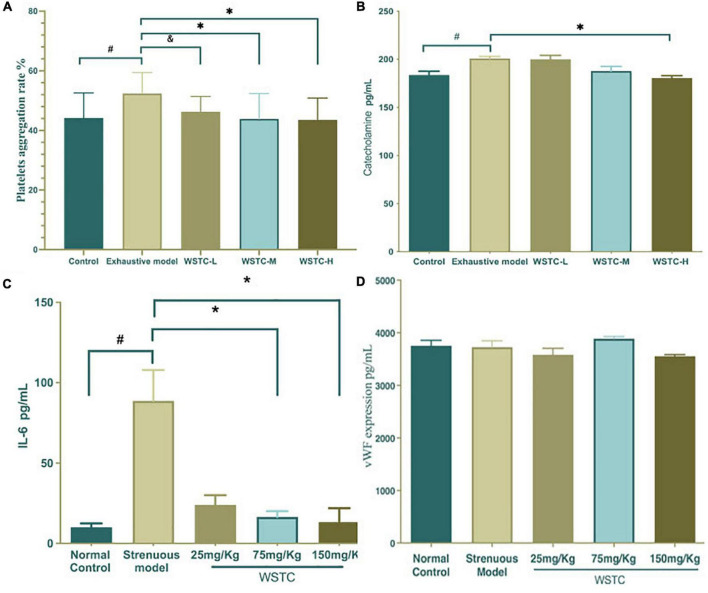
Effects of water-soluble tomato concentrate (WSTC) on platelet aggregation and the plasma levels of catecholamine and vWF in rats undergoing strenuous exercise. **(A)** Mean platelet aggregation rate of the control group, strenuous model group, WSTC 25 mg⋅kg^–1^ group, WSTC 75 mg⋅kg^–1^ group, and WSTC 150 mg⋅kg^–1^ group. All groups recorded the maximal aggregation after the addition of ADP (2.5 μM). ^#^*P* < 0.05 compared to control group; ^&^*P* < 0.10 compared to exhausted e model group; **(B)** Levels of plasma catecholamine in the control group, strenuous model group, WSTC 25 mg⋅kg^–1^ group, WSTC 75 mg⋅kg^–1^ group, and WSTC 150 mg⋅kg^–1^ group. ^#^*P* < 0.05 compared to control group; *P < 0.05, compared to strenuous model group. **(C)** Levels of plasma IL-6 in the control group, strenuous model group, WSTC 25 mg⋅kg^–1^ group, WSTC 75 mg⋅kg^–1^ group, and WSTC 150 mg⋅kg^–1^ group. ^#^*P* < 0.05 compared to control group; *P < 0.05, compared to strenuous model group. **(D)** Levels of plasma vWF in the control group, strenuous model group, WSTC 25 mg⋅kg^–1^ group, WSTC 75 mg⋅kg^–1^ group, and WSTC 150 mg⋅kg^–1^ group.

Compared with the normal control group, the maximum platelet aggregation rate in the strenuous exercise group was significantly higher (52.38 ± 7.03% *vs.* 44.19 ± 8.39%) (*P* < 0.05). The effects of platelet aggregation after 4 weeks of WSTC oral consumption in strenuous rats are shown in Figure 3A; an intake of 75 and 150 mg⋅kg^–1^ of WSTC significantly inhibited platelet aggregation *ex vivo* when compared with the strenuous model group (*P* < 0.01) with mean inhibition rates of 16.23 and 16.90%, respectively. FIB, TT, APTT, and PT assays demonstrated that the coagulation function was not influenced by WSTC (Supplementary Table 2).

Plasma catecholamine levels in the strenuous rat model were significantly higher than in the normal control group (200.88 ± 6.27 vs. 183.77 ± 10.86 pg⋅mL^–1^; *P* < 0.05). Compared with the strenuous exercise model, 150 mg⋅kg^–1^ of WSTC treatment significantly reduced the levels of catecholamine (180.56 ± 6.90 vs. 200.88 ± 6.27 pg⋅mL^–1^; *P* < 0.05) (Figure 3B). Plasma IL-6 levels in the strenuous rat model were higher than those in the normal control (80.70 ± 63.30 vs. 8.42 ± 8.06 pg⋅mL^–1^, *P* < 0.05). Compared with the strenuous exercise model, 75 mg⋅kg^–1^ and 150 mg⋅kg^–1^of WSTC treatment significantly reduced the levels of IL-6 (*P* < 0.05) (Figure 3C). However, the plasma levels of vWF were not significantly different when compared between all groups (*P* > 0.05) (Figure 3D).

### Effects of water-soluble tomato concentrate on thrombosis and platelet aggregation in the tails of model rats

**FIGURE 4:**
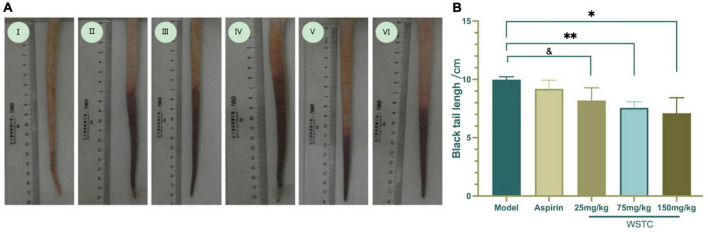
Effects of water-soluble tomato concentrate (WSTC) on rat tail thrombosis in the κcarrageenan-induced black tail model. **(A)** Representative images showing the tails of rats in the control group (I); κ-carrageenan-induced black tails in the model group (II); Aspirin group (III); WSTC 25 mg⋅kg^–1^ group (IV); WSTC 75 mg⋅kg^–1^ group (V), and WSTC 150 mg⋅kg^–1^ group (VI). **(B)** Mean length of black coloration in the tails of rats in the Model group; Aspirin group; WSTC 25 mg⋅kg^–1^ group, WSTC 75 mg⋅kg^–1^ group, and WSTC 150 mg⋅kg^–1^ group. ^&^*P* < 0.10 compared to model group. *P < 0.05, compared to model group; ^**^P < 0.01, compared to model group.

The length of the thrombus (as indicated by black tails) was measured at 2, 6, 24, and 48 h after κ-carrageenan injection. In the model group, black tails were observed at 2 h after injection; the extent of the black coloration extended to 10.35 ± 3.39 cm at 6 h. As time went on, the length of black coloration in the tails recovered slightly to 9.83 ± 3.28 cm and 9.79 ± 3.17 cm at 24 h and 48 h, respectively. Repeated measurement analysis of variance was used to analyze the changes in the length of black coloration in each treatment group over time. Compared with the model group, WSTC treatment, with the dose of 75 mg⋅kg^–1^ and 150 mg⋅kg^–1^ significantly inhibited the length of black coloration in the tail (*P* < 0.05), as shown in Figure 4B. Data relating to the length of black coloration in the tails of experimental rats in different treatment groups at different time points are shown in Table 1. Images of the black tails in each group 24 h after carrageenan injection are shown in Figure 4A.

**TABLE 1:** κ-carrageenan injection induced the black rat tail length at different time points with different treatment/cm (*$\bar{x}$* ± *s*).

		Time points after κ-carrageenan injection			
Group	Number	2 h	6 h	24 h	48 h
Control	9	0	0	0	0
Model	11	9.96 ± 3.90	10.35 ± 3.39	9.83 ± 3.28	9.79 ± 3.17
Aspirin	10	10.25 ± 4.82	9.13 ± 2.66	8.82 ± 3.26	8.58 ± 3.91
WSTC 25 mg⋅kg^–1^	10	8.70 ± 6.11	9.48 ± 5.03	7.42 ± 5.05	7.17 ± 5.23
WSTC 75 mg⋅kg^–1^	10	7.59 ± 3.16	8.26 ± 3.16	7.38 ± 3.78	7.00 ± 4.63
WSTC 150 mg⋅kg^–1^	10	8.91 ± 4.13	7.18 ± 3.21	6.50 ± 3.51	5.82 ± 4.38

**FIGURE 5:**
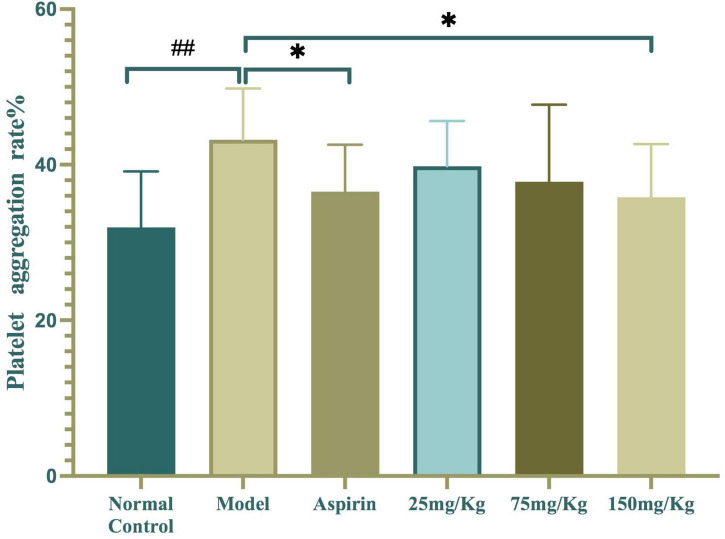
Effects of water-soluble tomato concentrate (WSTC) on platelet aggregation in the κcarrageenan-induced black tail model. Mean platelet aggregation rate in the normal control group, black tail model group, Aspirin group, WSTC 25 mg⋅kg^–1^ group, WSTC 75 mg⋅kg^–1^ group, and WSTC 150 mg⋅kg^–1^ group. All groups recorded the maximal aggregation rate after the addition of ADP (2.5 μM). ^##^*P* < 0.01 compared to normal group; *P < 0.05, compared to model group.

The inhibitory effect of WSTC on platelet aggregation *ex vivo* is shown in Figure 5. The injection of κ-carrageenan resulted in a higher platelet aggregation rate than the normal control with an aggregation rate of 43.2 ± 6.59% vs. 31.93 ± 7.22%, respectively (*P* < 0.01). Furthermore, WSTC treatment, at the dose of 150 mg⋅kg^–1^, inhibited platelet aggregation significantly with an inhibition rate of 15.42% (*P* < 0.01). Aspirin (30 mg⋅kg^–1^), a reference drug widely used as a platelet anti-aggregating agent in clinical practice, also significantly inhibited platelet aggregation in the tail thrombosis model, with an inhibition rate of 17.11%. The oral administration of WSTC at the dose of 75 mg⋅kg^–1^ showed a tendency (*P* < 0.10) to inhibit platelet aggregation. These results suggested that WSTC inhibited platelet activation in the inflammatory thrombosis model.

### Influences of water-soluble tomato concentrate on microcirculation perfusion and blood flow in tail thrombosis rats

We observed femoral artery blood flow and microcirculation perfusion in fixed regions of the hind limb, plantar, and tail of rats when anesthetized with 1.0% pentobarbital sodium (50 mg⋅kg^–1^). Compared with the control group, femoral artery blood flow and velocity were significantly increased in the model group (*P* < 0.05 or *P* < 0.01); there was also a tendency for an increase in blood flow shear rate (*P* = 0.07). Treatment with WSTC at the dose of 150 mg⋅kg^–1^ led to a significant reduction of the pulsatility index (PI) and resistance index (RI) significantly (*P* < 0.05) (Supplementary Table 3).

**FIGURE 6:**
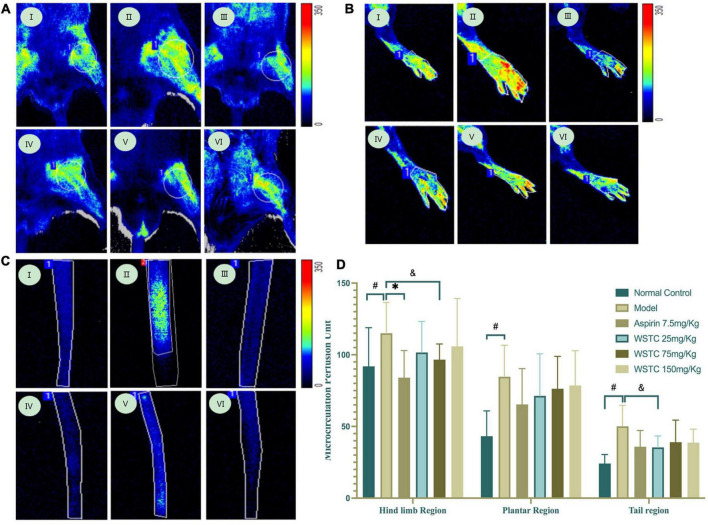
Effects of water-soluble tomato concentrate (WSTC) on microcirculation perfusion and blood flow in rats with tail thrombosis. **(A)** Representative images showing microcirculation perfusion in the hind limb region in control group (I); model group (II); Aspirin group (III); WSTC 25 mg⋅kg^–1^ group (IV); WSTC 75 mg⋅kg^–1^ group (V); and WSTC 150 mg⋅kg^–1^ group (VI). **(B)** Representative images showing microcirculation perfusion in the plantar region in the same order as **(A)**. **(C)** Representative images showing microcirculation perfusion at the tail region in the same order as **(A)**. **(D)** Mean microcirculation blood perfusion in the hind limb region, plantar region, and tail region. ^#^*P* < 0.05, compared to control group; *P < 0.05 compared to model group; ^&^*P* < 0.10, compared to model group.

Microcirculation blood perfusions in fixed regions of the hind limb, plantar, and tail were 91.88 ± 27.00 perfusion units (PU), 43.23 ± 17.64 PU, and 24.17 ± 6.26 PU, respectively, in the tail thrombosis model group; these values were significantly higher than those in the control group. Aspirin treatment significantly reduced the mean blood perfusion in the hind limb region (*P* < 0.01). Furthermore, WSTC at doses of 25 and 75 mg⋅kg^–1^ WSTC treatment led to reduced mean blood perfusion in the tail region (*P* = 0.09, *P* = 0.07) (Figure 6). These data implied that WSTC can facilitate the maintenance of healthy blood flow.

### Histological investigation of tail thrombosis after water-soluble tomato concentrate treatment

**FIGURE 7:**
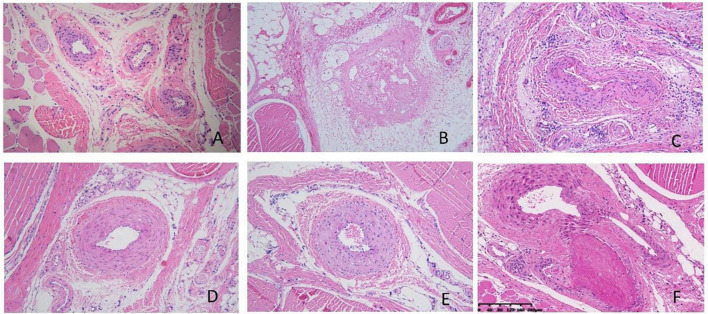
Pathological images of tail tissue under different treatments. **(A)** Normal control group, no defects in the intima of the vessel, the structure was normal and the vessel wall showed no abnormalities. **(B)** Model group, inflammation occurred with significant inflammatory cell infiltration and edema around the vessels, the vessels were blocked with mixed thrombi. Vessel walls were necrotic and showed homogeneous red staining. **(C)** Aspirin group; **(D)** water-soluble tomato concentrate (WSTC) 150 mg⋅kg^–1^ group, **(E)** WSTC 75 mg⋅kg^–1^ group, and **(F)** WSTC 25 mg⋅kg^–1^ group. The scale bar is 200 μm.

Pathological results showed that the injection of κ-carrageenan resulted in the formation of thrombosis in the caudal vein and caudal artery. We also observed perivascular inflammatory reactions with massive inflammatory cell infiltration and edema. Necrosis of the vessel walls and ischemic infarction of the tail with extensive tissue necrosis were evident in some animals. The administration of aspirin significantly alleviated caudal intravascular thrombosis, tissue anemia infarction, and edema. WSTC treatment at doses of 75 mg⋅kg^–1^ and 150 mg⋅kg^–1^ effectively alleviated caudal intravascular thrombosis, tissue anemia infarction, and tissue edema (*P* < 0.05) (Figure 7 and Supplementary Tables 4, 5).

## Discussion

In this study, the total amount of adenosine, chlorogenic acid, and rutin was 24 mg⋅kg^–1^ in WSTC. And previous quality control study showed that 6–10% (up to 9 mg) are known nucleoside derivatives (including adenosine, cytidine, guanosine, AMP, GMP, and deoxy derivatives), 13–15% (up to 10 mg) are known phenolic conjugates (e.g., caffeic and ferulic acids, chlorogenic acid, glycosides, and conjugates with quinic acid), and 8–10% (up to 7 mg) are known flavonoid derivatives, including a minimum of 2.4 mg quercetin derivatives per dose (1, 19).

Under high shear stress conditions, platelet adhesion depends on the interaction between vWF and the platelet receptor glycoprotein Ib (GP Ib), and this interaction is the only one able to resist the high shear rates that prevail in the microcirculation or stenosed arteries (26). vWF is a macromolecular soluble polysaccharide-protein stored in Weibel Palade bodies of endothelial cells, megakaryocytes, as well as platelet α granule, the A1 domain of vWF predominantly recognizes integrin GP Ibα receptors on platelets and mediates their binding. We found that WSTC inhibited platelet aggregation at shear rates of 8,000 s^–1^ and 4,000 s^–1^ (*P* < 0.05 or *P* < 0.01). WSTC showed inhibitory effects on vWF binding and SIPA at concentrations of 0.7, 2.0, and 6.0 g⋅L^–1^ under experimental conditions. At a high shear rate of 6,000 s^–1^, WSTC reduced the platelet adhesion rate and increased the rolling speed of platelets by inhibiting the binding of membrane integrin GP Ib to vWF protein. High pathological shear stress and abnormal flow patterns (such as recirculation at the bifurcation of blood vessels) can directly induce platelet activation, thus leading to aggregation and thrombosis. Therefore, the inhibition of SIPA is of great significance in maintaining normal blood flow.

A shear-induced *in vitro* platelet function test can assess clinically relevant anti-thrombotic effects (27). The inhibitive effects of tetramethylpyrazine and salvianolic acid B on SIPA were investigated by HAAKE rheometer RS 600 in Sprague-Dawley rats *in vitro* (28). Here, we employed a microfluidic system to generate different levels of shear stress to evaluate the effects of WSTC on SIPA *in vitro* and Sprague-Dawley rats were used as blood donors. Strenuous exercise-induced high shear stress and flow disturbances which can cause platelet aggregation and the development of an arterial thrombus, associated with an increased risk of vascular thrombotic events (29). Strenuous exercise promotes the extent of SIPA, possibly by enhancing the conformational change of vWF to bind to platelets and the subsequent activation of GP Ib-IX and GP IIb/IIIa complexes, as well as the expression of P-selectin in response to shear stress (30). It has been estimated that individuals undergoing strenuous exercise have an approximately 100-fold increase in the risk of a heart attack (31). Here, we used a strenuous exercise rat model to detect the platelet aggregation *ex vivo* and the corresponding inflammatory reaction under high shear forces. Shear stress of strenuous exercise depends on the intensity and type of exercise, and some studies showed that strenuous exercise can increase platelet sensitivity to ADP (32). This present study also revealed that the platelets of rats undergoing strenuous exercise appeared to be hyper-reactive and aggregated when compared with the rats in the normal control group. The oral consumption of WSTC for 4 weeks reduced the rats’ platelet aggregation by 16.23 and 16.90%, respectively, at doses of 75 and 150 mg⋅kg^–1^. We also found that high blood flow shear stress induced by strenuous exercise might not influence the levels of plasma vWF but do induce the conformational change of vWF, thus exposing its effective domain (33–35). Consequently, WSTC might affect platelet hyperactivity and block the binding of vWF to GP Ib.

Platelets are mechanical devices (36). The pathological low or high shear stress may influence the interplay between endothelial cells and platelets, which contribute to the prothrombotic state. The pathologically high shear environment can lead to occlusive thrombosis by SIPA from the interaction of platelets and vWF *via* glycoprotein Ib binding, following with P-selectin exposure (37). Age-related reduction of blood flow may result in atherosclerotic plaque erosion or stagnant blood flow. Blood flow reduction activates endothelial cells and results in the release of Weibel-Palade bodies containing vWF and P-selectin. These adhesion molecules trigger the inflammatory response, characterized by a complex interplay between platelets, leukocytes, and endothelial cells. A previous study showed that the WSTC supplementation for 4 weeks could moderately reduce platelet activation, aggregation, and granule secretion in older individuals with low shear rates (38). Together with our study, both represented a similar opinion of maintaining normal platelet aggregation which is good for healthy blood flow and vice versa.

Platelet-vWF interactions are also involved in inflammation (26). When vWF strings recruit platelets *via* interactions with the platelet receptor GP Ibα, a portion of these adhered platelets are activated with P-selectin exposure and interaction with PSGL-1 on rolling leukocytes under conditions of high shear rates (37). Neutrophil extracellular traps (NETs) are an essential interface between thrombosis and inflammation. The extracellular DNA backbone of NETs binds vWF, providing a substrate for platelet adhesion and thereby fostering their aggregation (39). Platelet activation provides a critical link between thrombosis and inflammation. The consequence of platelet activation during exercise increased inflammation, manifesting as increased levels of catecholamine and IL-6 (12). In the thrombosis process, plaque rupture and rapid platelet recruitment could be mediated by vWF-GP Ibα and GPVI-collagen interactions, a mutually activating interplay between platelets, neutrophils, and eosinophils, mediated by adhesion molecules (40, 41). Jong Shyan revealed that the increased levels of SIPA during high-intensity exercise may be related to the increased endogenous release of catecholamine and that strenuous exercise promoted the release of catecholamine (42). Subsequent research showed that catecholamine enhanced IL-6 release to levels sufficient to promote thrombosis (43). Catestatin, a catecholamine-release inhibitory peptide, was previously shown to inhibit the formation of thrombosis by attenuating endothelial inflammation by inhibiting the TLR4-p38 pathway (44). Based on the identification of an intimate association between inflammation and thrombosis, targeting the interface between these processes to prevent thrombosis seems highly promising (45, 46). In our present study, we found that WSTC reduced the plasma levels of catecholamine and IL-6 in rats undergoing strenuous exercise and that this strategy had potential as an intervention for inflammation.

To further investigate the effects of WSTC on inflammation and platelet hyperactivity, the use of the carrageenan-induced rat tail thrombosis model (47) may help to identify the impact of WSTC on inflammation-induced platelet hyperactivity. It was previously reported that stasis and turbulence in the blood flow are effective factors for generating this type of thrombosis model (16). Our experiments showed that dietary WSTC can potentially reduce platelet hyperactivity during an inflammatory response and help to maintain a healthy blood flow. In the model group, black tails appeared 2 h after the injection of κ-carrageenan; a dark gray color was also observed in the paw, ear, and perioral area of some rats, indicating local microcirculation disorders and the formation of micro-thrombi. However, approximately 6 h after κ-carrageenan injection, except for the black tail, other regions showing a dark color or microcirculation disturbance gradually disappeared. The length of the black coloration of rats in the WSTC treatment groups was obviously shorter than those in the model group, thus indicating the antithrombotic effects of oral WSTC in this rat model of tail thrombosis. Platelet thrombosis in the rat tail artery and inflammatory cell infiltration around the artery were observed by pathological tissue analysis. Compared with the model group, the extent of thrombosis and inflammation were reduced following WSTC treatment at doses of 75 and 150 mg⋅kg^–1^.

Platelet dysfunction during inflammation is also reflected by exhibiting an increased sensitivity to endogenous agonists such as collagen and ADP (48). Sulforaphane, a naturally occurring isothiocyanate, has been shown to display both anti-inflammatory and anti-thrombotic actions in cerebral microcirculation (49). Indole-3-carbinol (I3C), a phytochemical compound of cruciferous vegetables affects inflammatory processes within the microcirculation, including thrombus formation and microvascular leakage (50). The administration of clopidogrel in healthy persons with high baseline platelet aggregation results in improved thermal hyperemia-induced microcirculatory endothelial function, which suggests that clopidogrel may have a beneficial effect on microcirculatory endothelial function, presumably through antiplatelet activity, and may confer additional vascular benefits (51).

Microcirculation plays a vital role in homeostatic tissue injury and inflammation (52). Inflammation is a microcirculation-dependent tissue response to extrinsic and intrinsic stimuli (53). During such an inflammatory response, the signs of inflammation that can be observed are heat, pain, redness, and swelling. In the early phase of an inflammatory response, hyperemia and alteration in tissue blood flow are often noted. The microcirculation can be most easily examined in the periphery including skin, retina, and limb vasculature (54). It was shown that the femoral blood flow increased during the administration of acetylcholine, indicative of resistance to arteriolar endothelial function (55). In our study, WSTC was shown to blunt inflammation and ameliorate microcirculation perfusion.

The femoral artery and middle iliac artery were derived from the common iliac artery in rats, which is close to the location of the tail thrombus; this allowed superficial access to observe the femoral artery. A direct blood supply was detected between the median artery and the femoral artery in the rat tail (56). In the present study, we used a small animal ultrasound instrument (vevo2100) to detect changes in blood flow in the femoral artery; then, we calculated blood flow and shear rate according to the measured inner diameter and blood flow velocity. Analysis showed that local thrombosis influenced systolic and diastolic femoral artery blood flow in the model group. The resistance index (RI) and pulsatility index (PI) are indices that reflect blood flow resistance in the arteries. A reduction in PI represents an increase in diastolic blood flow and a decrease in vascular resistance, while a reduction in RI means a decrease in distal vascular resistance. Our results demonstrated that WSTC at a dose of 150 mg⋅kg^–1^ significantly reduced PI and RI compared with the model group, thus implying that WSTC may improve blood flow and help to prevent cardio-cerebrovascular diseases. κ-carrageenan-induced thrombosis was accompanied by platelet hyperactivity along with a reduction in regional blood flow and microcirculation perfusion; these events may have been related to inflammatory reactions. The oral administration of WSTC ameliorated deleterious effects and helped to maintain normal microcirculation perfusion.

## Conclusion

For the first time, we showed that WSTC can inhibit SIPA by affecting binding between integrin GP Ib and vWF *in vitro.* We also demonstrated the beneficial effects of WSTC on platelet hyperactivity resulting from strenuous exercise and inflammation by maintaining normal blood flow and microcirculation *in vivo*.

## Data availability statement

The raw data supporting the conclusions of this article will be made available by the authors, without undue reservation.

## Ethics statement

The animal study was reviewed and approved by the Animal Care and Use Committee of Institute of Chinese Materia Medica, China Academy of Chinese Medical Sciences.

## Author contributions

YY and XZ contributed to the conception and design of the study. LuL, SX, LeL, ZS, QZ, and XY performed the experiments. LuL, SX, YlW, YfW, and FL analyzed and interpreted the data. YY and LuL wrote the manuscript. All authors contributed to the manuscript revision and approved the submission.
