# Supplementary material for: Water-soluble tomato concentrate modulates shear-induced platelet aggregation and blood flow in vitro and in vivo
Source: Front Nutr. 2022 Sep 2;9:961301. doi: 10.3389/fnut.2022.961301 (PMC9478107; doi:10.3389/fnut.2022.961301)
Supplement: Supplementary file 1 [file Data_Sheet_1.docx]

Supplementary Material

**Supplementary material**


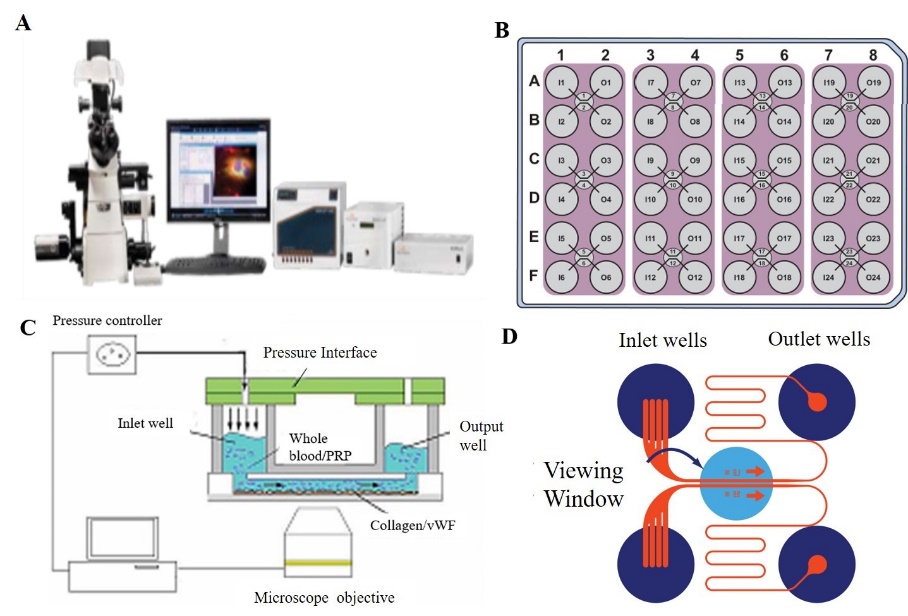
**Supplementary Figure 1 The structure of the microfluidics system**

Supplementary Figure 1: The Bioflux1000Z Microfluidic Culture System. (A) Bioflux1000Z Instrument components. From left to right are the automated Zeiss microscope with the CCD camera, PC Workstation, BioFlux Controller and Fluorescence Module. (B) BioFlux Plate. This plate features 24 experimental channels, each with an input and output well. (C) Principles of Operation. The flow channels can be coated with proteins, adhesion molecules and cellular monolayers to conduct a wide range of cell-cell and cell-ligand interaction assays. (D) Viewing Window.

**Supplementary Table 1 Body weight of rats（‾*x*±*s*）**

| Group | Initial | First week (g) | Second week (g) | Third week (g) | Fourth week (g) |
| --- | --- | --- | --- | --- | --- |
| Control | 173.13 ± 5.30 | 244.09 ± 9.29 | 285.00 ± 13.22 | 297.27 ± 15.13 | 305.00 ± 22.64 |
| Model | 174.70 ± 12.72 | 240.73 ± 12.87 | 263.82 ± 21.45 | 278.82 ± 30.00 | 281.27 ± 38.44 |
| Aspirin | 175.49 ± 9.07 | 240.00 ± 11.32 | 275.73± 20.28 | 287.36 ± 25.54 | 296.36 ± 31.73 |
| WSTC 25 mg·kg-1 | 174.90 ± 11.13 | 240.55 ± 10.83 | 268.09 ± 12.28 | 277.64 ± 24.06 | 278.73 ± 30.28 |
| WSTC 75 mg·kg-1 | 174.16 ± 7.80 | 237.18 ± 11.91 | 274.64 ± 14.78 | 302.18 ± 18.62 | 314.09 ± 20.10 |
| WSTC 150 mg·kg-1 | 172.95 ± 8.94 | 237.45 ± 11.80 | 264.18 ± 17.59 | 289.45 ± 27.55 | 299.27 ± 32.32 |

**Supplementary Table 2 Effects of WSTC on coagulation function in strenuous exercise rats（‾*x*±*s*）**

| Group | APTT (Sec) | PT (Sec) | TT (Sec) | FIB (g·L^-1^) |
| --- | --- | --- | --- | --- |
| Control | 25.75 ± 2.03 | 13.27 ± 0.98 | 37.48 ± 1.45 | 4.08 ± 0.36 |
| Model | 27.21 ± 2.04 | 13.97 ± 1.57 | 36.09 ± 0.95 | 4.22 ± 0.23 |
| WSTC 25 mg·kg^-1^ | 26.48 ± 1.97 | 13.18 ± 0.82 | 36.65 ± 0.70 | 4.05 ± 0.34 |
| WSTC 75 mg·kg^-1^ | 28.09 ± 1.99 | 13.94 ± 1.20 | 36.61 ± 1.47 | 4.10 ± 0.29 |
| WSTC 150 mg·kg^-1^ | 27.69 ± 1.51 | 13.89 ± 0.57 | 37.15 ± 0.91 | 4.15 ± 0.33 |

**Supplementary Table 3 Effects of WSTC on femoral artery blood flow in tail thrombosis rats（‾*x*±*s*）**

| Group | D  /mm | EDV  /mm·s^-1^ | PSV  /mm·s^-1^ | MV  /mm·s^-1^ | PI | RI | Blood flow  /ml·min^-1^ | Shear rate  /s^-1^ |
| --- | --- | --- | --- | --- | --- | --- | --- | --- |
| Control | 0.28±0.05 | 24.00±11.22 | 155.15±32.53 | 66.43±14.72 | 1.61±0.32 | 0.73±0.06 | 64.51±14.38 | 2012.7±653.29 |
| Model | 0.31±0.04 | 44.84±17.71^#^ | 225.61±59.10^##^ | 98.83±22.82^##^ | 1.82±0.38 | 0.79±0.08 | 120.86±35.05^##^ | 2639.94±780.59 |
| Aspirin | 0.31±0.03 | 39.18±19.90 | 203.49±41.10 | 89.76±22.06 | 1.88±0.40 | 0.81±0.08 | 112.30±41.21 | 2350.06±545.01 |
| WSTC 25mg/Kg | 0.32±0.04 | 44.84±24.33 | 224.96±62.55 | 97.97±32.95 | 1.90±0.34 | 0.81±0.07 | 128.62±46.27 | 2511.30±879.47 |
| WSTC 75mg/Kg | 0.28±0.05 | 44.02±11.91 | 218.36±57.12 | 99.53±25.77 | 1.76±0.24 | 0.79±0.04 | 107.84±45.48 | 2682.51±817.06 |
| WSTC 150mg/Kg | 0.30±0.03 | 62.39±14.83^*^ | 228.43±51.54 | 112.15±21.87 | 1.47±0.23^*^ | 0.72±0.07^*^ | 139.58±50.33 | 2253.98±431.29 |

Supplementary table 3：Compared with normal control，^#^*P*<0.05，^##^*P*<0.01; Compared with model group；**P*<0.05，***P*<0.01

**Supplementary Table 4 Inflammation and thrombosis score on the rat tail vessels (H&E slides)**

| Histological change | Caudal artery intimal inflammation, atresia, thrombus in lumen, arterial wall necrosis | | | | | | Caudal vein intimal inflammation, infarction, thrombus in lumen, necrosis | | | | | | Perivascular tissue inflammation, edema, necrosis | | | | | |
| --- | --- | --- | --- | --- | --- | --- | --- | --- | --- | --- | --- | --- | --- | --- | --- | --- | --- | --- |
| **Score** | 0 | 1 | 2 | 3 | 4 | Wilcoxon test rank | 0 | 1 | 2 | 3 | 4 | Wilcoxon test rank | 0 | 1 | 2 | 3 | 4 | Wilcoxon test rank |
|  |  |  |  |  |  |  |  |  |  |  |  |  |  |  |  |  |  |  |
| **Control** | 7/8 | 0/8 | 1/8 | 0/8 | 0/8 | 9.63 | 8/8 | 0/8 | 0/8 | 0/8 | 0/8 | 9.50 | 3/8 | 2/8 | 3/8 | 0/8 | 0/8 | 13.00 |
| **Model** | 0/8 | 0/8 | 1/8 | 1/8 | 6/8 | 41.38^##^ | 1/8 | 0/8 | 0/8 | 2/8 | 5/8 | 38.38^##^ | 0/8 | 0/8 | 0/8 | 1/8 | 7/8 | 43.06^##^ |
| **Aspirin** | 0/8 | 5/8 | 0/8 | 3/8 | 0/8 | 25.38^**^ | 1/8 | 0/8 | 2/8 | 5/8 | 0/8 | 29.31^**^ | 0/8 | 0/8 | 3/8 | 5/8 | 0/8 | 28.88^**^ |
| **WSTC 25mg/Kg** | 1/8 | 1/8 | 3/8 | 2/8 | 1/8 | 28.31^**^ | 1/8 | 2/8 | 1/8 | 4/8 | 0/8 | 26.94^**^ | 2/8 | 2/8 | 0/8 | 4/8 | 0/8 | 20.75^**^ |
| **WSTC 75mg/Kg** | 3/8 | 3/8 | 1/8 | 1/8 | 0/8 | 17.75^**^ | 5/8 | 0/8 | 1/8 | 2/8 | 0/8 | 17.81^**^ | 0/8 | 5/8 | 1/8 | 2/8 | 0/8 | 19.13^**^ |
| **WSTC 150mg/Kg** | 2/8 | 2/8 | 1/8 | 2/8 | 1/8 | 24.56^**^ | 2/8 | 2/8 | 1/8 | 2/8 | 1/8 | 25.06^**^ | 2/8 | 2/8 | 0/8 | 3/8 | 1/8 | 22.19^**^ |

Supplementary table 4: Non-parametrical test (Wilcoxon rank sum test) was used to detect the difference among the 6 groups. n=8 in each group.

Score system: see Supplementary table 5

## *P*<0.01 compared with the control group, ** *P*<0.01 compared with the model group

**Supplementary table 5 Histological grade and score system on rat tail thrombosis and inflammation**

| Histological grade | **Score** | Vascular histological changes | | |
| --- | --- | --- | --- | --- |
|  |  | Caudal artery | Caudal vein | Perivascular tissue |
| - | **0** | The arterial wall and lumen is normal, and intima is intact | The vein lumen is normal, and intima is intact | No inflammatory and edema was seen in perivascular tissue |
| + | **1** | mild endometritis and mild atresia, artery thrombosis occlusion <25%. | mild vein endarteritis, vein thrombosis occlusion <25% | Very mild inflammatory cell infiltration and slight edema |
| ++ | **2** | Moderate intima inflammation and atresia, thrombus obstruction area of 25%~50% artery lumen | Moderate intima inflammation and atresia, thrombus obstruction area of 25%~50% vein lumen | Mild inflammatory cell infiltration and mild edema |
| +++ | **3** | Severe intima inflammation and atresia, thrombus obstruction area of 50%~75% artery lumen | Severe intima inflammation and atresia, thrombus obstruction area of 50%~75% vein lumen | A large area of diffuse inflammatory reaction and moderate edema |
| ++++ | **4** | The caudal artery is severely to completely atresia, thrombus occluded almost the whole artery lumen, the arterial wall is necrotic. | The caudal vein is severely to completely atresia, thrombus occluded almost the whole lumen, vein is necrotic. | A large area of necrosis in the perivascular tissue, the tissue structure almost disappeared with severe edema |
